# Correlation between aortic/carotid atherosclerotic plaques and cerebral infarction

**DOI:** 10.3892/etm.2013.1129

**Published:** 2013-05-23

**Authors:** BAOJUN WANG, SHAOLI SUN, GUORONG LIU, YUECHUN LI, JIANGXIA PANG, JINGFEN ZHANG, LIJUAN YANG, RUIMING LI, HUI ZHANG, CHANGCHUN JIANG, XIUE LI

**Affiliations:** 1 Department of Neurology, Baotou Central Hospital, Baotou, Inner Mongolia 014040;; 2 Department of Cadre Healthcare, Affiliated Yuhuangding Hospital of Qingdao University, Yantai, Shandong 264000, P.R. China

**Keywords:** aortic atherosclerotic plaque, carotid atherosclerotic plaque, cerebral infarction

## Abstract

The aim of this study was to investigate the correlation between aortic/carotid atherosclerotic plaques and cerebral infarction. We examined 116 cases of cerebral infarction using transcranial Doppler ultrasound in order to exclude cerebrovascular stenosis. Transesophageal echocardiography and color Doppler ultrasound were used to detect aortic atherosclerotic plaques (AAPs) and carotid atherosclerotic plaques (CAPs). AAPs were detected in a total of 70 of the 116 cases (60.3%), including 56 with moderate/severe atherosclerotic changes (48.3%). The difference in the incidence of various types of infarction between APP severity levels was significant (P<0.01). Of the 116 cases, 64 had CAPs (55.2%), including 46 with unstable plaque (39.7%). The difference in the incidence of various types of infarction between CAP stability levels was significant (P<0.01). The results indicate that moderate/severe AAP and unstable CAP are significant causes of embolic infarction without stenosis in the internal carotid arteries.

## Introduction

Stroke is the third most common cause of disability worldwide and the third leading cause of mortality; of these mortalities, 67.3–80.5% are the result of an ischemic stroke (1). A previous study revealed that the incidence of ischemic stroke is rising annually at a rate of 8.7% in China (2). Of these cases, ∼30% are fatal and 70% of the survivors have hemiplegia, aphasia and other disabilities, which impose a heavy burden on society and the families of the patients. Atherosclerosis is a major cause of ischemic stroke, and is significant in its development. Extracranial embolism causes ∼50% of all cases of ischemic stroke. Although carotid artery plaques and cardiogenic emboli have been observed as the embolic sources in patients with cerebral infarction, ∼1/3 of embolic sources remain unknown (3). Severe aortic arch atherosclerotic (AAA) plaques may be detected in ischemic stroke patients for which no embolic source has been identified; in addition, an unexplained cerebral embolism is considered to be associated with AAA (4–7). Yahia *et al* have demonstrated the importance of performing early examination by transesophageal echocardiography (TEE) in patients who have had an unexplained stroke (8).

In the current study, patients with cerebral infarction underwent TEE and heart valve examinations. All patients underwent transcranial Doppler (TCD) examination in order to exclude cerebrovascular stenosis upon enrollment in the study. TEE and color Doppler ultrasound were used to detect aortic atherosclerotic plaques (AAPs) and carotid atherosclerotic plaques (CAPs), in order to investigate the correlation between AAPs/CAPs and cerebral infarction.

## Subjects and methods

### Clinical data

A total of 116 patients with cerebral infarction were selected (32–74 years old; 78 males, 38 females). The patients had been hospitalized between August 2005 and December 2008 in the Neurology Department of Baotou Central Hospital, Inner Mongolia Autonomous Region, China. All patients had symptoms and signs of nervous system alterations such as hemiplegia or aphasia, confirmed by brain CT or MRI. The levels of muscle strength were graded between 0 and 4. Of the 116 patients, 59 had hypertension, 29 had hyperlipidemia, 36 had diabetes and 43 had hyperhomocysteinemia. The heart valves, heart cavity with or without mural thrombus, ascending aorta, aortic arch and descending aorta of all patients were assessed using transesophageal ultrasound. CAPs were examined using color Doppler ultrasound, and intracranial hemodynamics were determined by TCD ultrasound. The exclusion criteria were as follows: i) history of heart disease, heart valve disease and intracardiac mural thrombus; ii) moyamoya disease, Takayasu’s arteritis and embolism; iii) intracranial vascular stenosis examined by TCD ultrasound; iv) massive cerebral infarction; and v) blood system diseases. This study was conducted in accordance with the Declaration of Helsinki and was approved by the Ethics Committee of Baotou Central Hospital. All patients provided written informed consent.

### Diagnostic criteria

A lacunar cerebral infarction has an infarct diameter of ≤15 mm. Small/medium-sized areas of infarction refer to an infarct between the lacunar cerebral and large cerebral infarction. Multiple cerebral infarctions referred to multiple ischemic infarct in brain. In total, lacunar cerebral infarctions were identified in the basal ganglia in 48 cases and in the pontine area in 10 cases; small/medium-sized areas of infarction were noted in 5 cases in the temporal lobe, 12 in the parietal lobe, 7 in the frontal lobe and 2 in the cerebellum; and 32 cases had multiple cerebral infarctions in the basal ganglia, temporal lobe, parietal lobe and cerebellum. Of the 32 cases of multiple cerebral infarctions, 20 were acute multiple cerebral infarctions, confirmed by NMR diffusion imaging.

### Ultrasound examination

All patients diagnosed by a neurologist were examined with TCD, color Doppler ultrasound and TEE using a GE Vivid3 dual-function color ultrasound diagnostic apparatus (GE Instruments Inc., Aurora, OH, USA) with a 3.5 MHz esophageal probe.

### AAP grading

The following criteria were used for AAP grading: normal, the aortic inner wall was smooth and complete; mild, <1.0 mm of mildly thickened intima; moderate, 1.0–3.9 mm of moderately thickened intima; and severe, ≥4 mm of irregular or active atherosclerotic plaque was prominent in the lumen.

### Statistical analysis

Statistical significance was determined using the χ^2^ test for χ-list data using SPSS software version 12.0 (SPSS, Inc., Chicago, IL, USA). P<0.05 was considered to indicate a statistically significant difference.

## Results

### TEE

AAP lesions were observed in 70 cases of cerebral infarction (Table I). Plaques in the ascending aorta, aortic arch and descending aorta accounted for 5.7% (n=4), 77.1% (n=54) and 17.1% (n=12) of the 70 AAP cases, respectively. The degree of severity of the atherosclerotic plaques differed between the sites. The atherosclerotic plaques in the four ascending aorta cases were graded as moderate. In the aortic arch, mild, moderate and severe changes to the aortic plaques were observed in 10, 24 and 20 cases, respectively, whereas mild, moderate and severe changes to aortic plaques in the descending aorta were observed in 4, 6 and 2 cases, respectively. Moderate/severe atherosclerotic changes were detected in 48.3% of the patients with cerebral infarction.

### Color Doppler ultrasound examination

Of all cases, CAPs and unstable plaques accounted for 55.2% (64/116) and 39.7% (46/116), respectively. Bilateral unstable plaques were observed in 28 cases, bilateral stable plaques in 10, unilateral unstable plaques in 18 and unilateral stable plaques in 8.

### TCD ultrasound examination

The TCD ultrasound examination did not identify any changes in the patients that were sufficient for diagnosis as vascular stenosis.

### Examination of patients with AAP and CAP

Of the 70 cases of AAP, 64 were accompanied by CAP. Of the 56 cases with moderate/severe AAP, 42 cases were accompanied by unstable CAP, and 14 cases were accompanied by stable CAP. Of the 14 cases with mild AAP, 8 cases were accompanied by CAP, including 4 cases with stable plaque and 4 cases with unstable plaque.

### Correlation between AAP or CAP and cerebral infarction

Of the patients with cerebral infarction, lacunar cerebral infarction, multiple cerebral infarction and small/medium areas of infarction were observed in 58, 32 and 26 cases, respectively.

Mild, moderate and severe AAP in patients with lacunar cerebral infarction were observed in 8, 8 and 5 cases, respectively; in patients with multiple cerebral infarctions they were observed in 4, 14 and 6 cases, respectively, and in patients with a small/medium area of infarction, they were observed in 2, 12 and 11 cases, respectively. The results of statistical analysis by χ^2^ test revealed that the difference in the incidence of various types of infarction between APP severity levels was significant (χ^2^=39.52, P<0.01; Table II). With normal or mild AAP, the incidence of lacunar cerebral infarction was higher than that of small/medium area infarction. The incidence of small/medium area infarction in patients with moderate/severe AAP was higher than in those with mild or normal AAP.

Stable and unstable CAPs in patients with lacunar cerebral infarctions were observed in 12 and 6 cases, respectively. Stable and unstable CAPs in patients with multiple cerebral infarctions were observed in 4 and 20 cases, respectively. Stable and unstable CAPs in patients with small/medium area of infarctions were observed in 2 and 20 cases, respectively. The results of statistical analysis by χ^2^ test revealed that the difference in the incidence of various types of infarction between the two CAP stability levels was significant (χ^2^=43.47, P<0.01; Table III). The incidence of embolic cerebral infarction in patients with unstable CAP is higher than in those with stable CAP.

## Discussion

In the current study, the rate of AAP incidence in patients with cerebral infarction was 60.3% (70/116). The site with the highest incidence of plaques was the AAA, at 77.1% (54/70). Moderate/severe changes in AAPs accounted for 48.3% of the patients with cerebral infarction (56/116). The risk of stroke increased in patients with severe aortic atherosclerosis, and the highest risk was observed in patients with plaques of ≥4 mm thickness. This finding is consistent with the results reported by Molisse *et al* (9) and Ueno *et al* (10). A study concerning aortic plaques and the risk of ischemic stroke in the USA reported that large aortic plaques correlated with an increased risk of ischemic stroke (11). Therefore, timely detection and diagnosis are of clinical significance for patients with moderate/severe AAP, as this facilitates the administration of preventive treatment, particularly for preventing the occurrence and recurrence of cerebral infarction.

The difference in the incidence of various types of infarction between APP severity levels was significant (χ^2^=39.52, P<0.01; Table II). The incidence of a small/medium area of infarction in patients with moderate/severe AAP was higher than that in patients with mild or normal AAP. This may be attributed to the facile shedding of moderate/severe AAP, leading to the formation of emboli and small/medium area infarction. With normal or mild AAP, the incidence of lacunar cerebral infarction was higher than that of a small/medium area of infarction. This may be attributed to the minute arteriosclerosis of deep brain-perforating branches caused by other factors such as hypertension. Furthermore, the incidence of multiple cerebral infarctions was higher than that of lacunar cerebral infarction in patients with moderate/severe AAP. This finding suggests that embolic shedding may be a significant cause of multiple cerebral infarctions. Di Tullio *et al* proposed that acute multiple cerebral infarctions were indicators of thromboembolism (12).

In the current study, unstable CAP accounted for 39.66% (46/116) of the patients with cerebral infarction. The difference in the incidence of various types of infarction between the two CAP stability levels was significant (χ^2^=43.47, P<0.01; Table III). The incidence of embolic cerebral infarction in patients with unstable CAP was higher than in those with stable CAP. Unstable CAP is significant in the formation of cerebral infarction (13–15). Patients with severe carotid stenosis or intracranial vascular stenosis were excluded from the current study. We hypothesize that unstable CAP leads to embolic shedding, which results in embolic infarction.

The results from the present study demonstrated that the incidence of AAP in patients with cerebral infarction was higher than that of CAP, suggesting the need to focus on the role of AAP in the development of acute stroke. Other active examinations, such as TEE, should be performed in order to identify risk factors for strokes and provide effective navigation for its treatment. The degree of severity of AAP may be inconsistent with that of CAP, which is considered to be a joint or separate source of emboli, resulting in the occurrence of embolic cerebral infarction. Furthermore, we demonstrated that moderate/severe AAP and unstable CAP are significant causes of embolic cerebral infarction.

## Figures and Tables

**Table I.** Incidence of atherosclerotic plaques of different degrees of severity at various sites.

Site	Mild	Moderate	Severe
Aortic arch	10	24	20
Descending aorta	4	6	2
Ascending aorta	0	4	0

**Table II.** Correlation between the degree of AAP and the type of infarction.

Degree of aortic atherosclerosis	Cases	Lacunar cerebral infarction	Multiple cerebral infarction	Small/medium area of infarction
Normal	46	37	8	1
Mild	14	8	4	2
Moderate	34	8	14	12
Severe	22	5	6	11

χ^2^=39.52, P<0.01. AAP, aortic atherosclerotic plaque.

**Table III.** Correlation between the degree of CAP and the type of infarction.

Degree of carotid atherosclerosis	Cases	Lacunar cerebral infarction	Multiple cerebral infarction	Small/medium area of infarction
Normal	52	40	8	4
Stable plaque	18	12	4	2
Unstable plaque	46	6	20	20

χ^2^=43.47, P<0.01. CAP, carotid atheroscleotic plaque.
